# Luteolin Suppresses the Proliferation of Gastric Cancer Cells and Acts in Synergy with Oxaliplatin

**DOI:** 10.1155/2020/9396512

**Published:** 2020-02-21

**Authors:** Li-Qun Ren, Qi Li, Yang Zhang

**Affiliations:** ^1^Department of Experimental Pharmacology and Toxicology, School of Pharmaceutical Sciences, Jilin University, Changchun, Jilin, China; ^2^Department of Pathology, The Third Affiliated Hospital of Jinzhou Medical University, Jinzhou, Liaoning, China

## Abstract

*Objective*. Gastric cancer, one of the most common malignant tumors worldwide, arises from the gastric mucosal epithelium and severely affects patient health and quality of life. Luteolin (LUT) is a flavonoid found in vegetables and fruits with diverse functions. A large number of studies have confirmed that LUT has an antitumor effect. Therefore, this study is aimed at verifying whether LUT can exert antitumor effects in synergy with oxaliplatin (OXA). As such, we examined the effects of LUT, OXA, and their coadministration in a gastric adenocarcinoma cell line (SGC-7901). We used the MTT assay to quantify the proliferation of SGC-7901 cells, flow cytometry to detect the cell cycle and apoptosis, ELISA to detect the expression of cell-cycle-related proteins, and western blot to detect the expression of related apoptotic factors. The results of this study show that the combination of LUT and OXA inhibited SGC-7901 cell proliferation and induced apoptosis by altering cell-cycle proportions. In addition, the combination also activated Cyt c/caspase signaling in SGC-7901 cells. In summary, LUT synergy with OXA inhibited the proliferation of gastric cancer cells *in vitro*. The present study also elucidated the mechanism by which LUT potentiated the sensitivity of SGC-7901 cells to OXA through the Cyt c/caspase pathway.

## 1. Introduction

**Figure 1:**
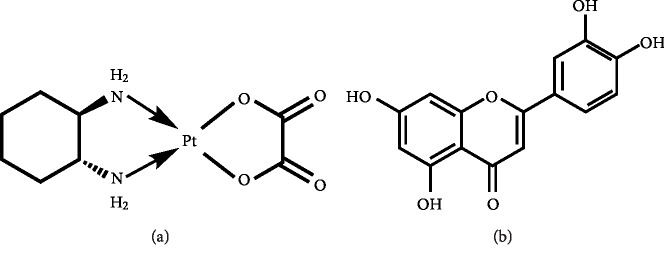
Chemical structure: (a) oxaliplatin; (b) luteolin.

Gastric cancer, one of the most common malignant tumors worldwide [1], arises from the gastric mucosal epithelium and severely affects patient health and quality of life. There are obvious regional differences in its occurrence, with Japan, Korea, and China being the areas with the highest risk in Asia [1, 2]. In particular, gastric cancer is one of the most prominent malignant tumors in China [3]. Gastric cancer has the fifth highest incidence rate and third highest mortality rate worldwide [4], causing a great burden on public health. Oxaliplatin (OXA, Figure 1(a)) belongs to the third generation of platinum compounds used as chemotherapeutic and is gradually becoming the primary drug for the treatment of advanced gastric cancer. Although chemotherapeutic regimens for gastric cancer [5] are continuously improving, adverse effects following gastric cancer chemotherapy still occur in some patients. In addition, resistance to chemotherapeutic drugs occurs or develops in most tumor cells, leading to treatment failure [6]. Consequently, the 5-year survival rate of patients with advanced gastric cancer has not increased significantly in recent years [7]. In addition, OXA generates high levels of oxidative stress [8] in cells, which leads to cell dysfunction and unpredictably impacts the cell. The method by which the efficacy of the drug would be optimized while also reducing its side effects is still being investigated.

The use of natural compounds found in food and plants for treating gastric cancer has been increasing in the past 20 years [9]. In recent years, a few studies identified therapeutic candidates, namely, quercetin [10], liquiritin [11], and kaempferol [12], for the treatment of gastric cancer via inhibition of cell proliferation and induction of apoptosis. Luteolin (LUT, Figure 1(b)) is a flavonoid which is abundantly found in vegetables and fruits such as celery, broccoli, carrots, and peppers [13]. LUT has various biological functions such as being anti-inflammatory [14], antiatherogenic [15], and having antitumor effects [16]. In addition, LUT was shown to reduce proliferation and induce apoptosis in human osteosarcoma cells by regulating the apoptotic proteins BCL-2, BAX, caspase-3, and survivin [17]. Nevertheless, the biological functions and intracellular mechanisms of LUT in gastric cancer have not yet been elucidated. Gastric adenocarcinoma is the most common type of gastric cancer [18].

In the current study, we examined the effects of LUT on gastric cancer SGC-7901 cells. The objectives of the current study were (a) to investigate the effect of LUT on proliferation and cell-cycle regulation in SGC-7901 cells, (b) to examine the apoptotic effect of LUT on the cells, (c) to investigate the intracellular signaling molecules controlled by LUT in gastric cancer, and (d) to confirm the synergistic effects of LUT and OXA in inhibiting the proliferation of SCG-7901 cells.

## 2. Materials and Methods

### 2.1. Chemicals and Reagents

OXA was purchased from Sigma-Aldrich (Massachusetts, USA), and LUT (purity > 98%) was purchased from Jingzhu Technology Co. Ltd., China. Methyl thiazolyl tetrazolium (MTT) was purchased from Sigma-Aldrich (Massachusetts, USA). The Hoechst33258 kit and Cell Cycle and Apoptosis Analysis Kit were purchased from the Beyotime Institute of Biotechnology (Nanjing, China). The Cyclin D1 ELISA Kit (ab214571), Cytochrome c (Cyt c), pro-caspase-3, cleaved-caspase-3, Bax, and Bcl-2 antibodies were purchased from Abcam (MA, USA). All the other reagents used were of the highest commercially available purity. LUT was dissolved in dimethyl sulfoxide (DMSO) to ensure the concentrations were lower than 0.1%.

### 2.2. Cell Culture

SGC-7901 cells were purchased from ATCC (Shanghai, China) and maintained in high glucose Dulbecco's modified Eagle's medium (DMEM) containing fetal bovine serum (10%), streptomycin (100 mg/mL), and penicillin G (70 mg/mL) at 37°C in a humidified atmosphere of 5% carbon dioxide and 95% air.

### 2.3. Pharmacological Interference

In order to determine the doses of LUT and OXA administered alone and in combination, the effect of different concentrations of LUT and OXA on the survival of SGC-7901 cells was measured using the MTT assay. As indicated, after 24 h of treatment, changes in cell morphology and density were observed. The medium-effect principle was used to calculate the drug concentration and the combined dose. The inhibition rate (fa) was calculated using the following formula: fa = 1‐(average optical density of the experimental group/average optical density of the control group), while the medium-effect was calculated using the equation fa/fu = (*D*/Dm). The intermediate concentration Dm of each drug, either alone or in combination, which is the basis for the grouping of drugs based on concentration and combined dose, was calculated. The combined index (CI) = D1 for various effects when the two drugs are combined (Dx1 + D2/Dx2 + *α*D1D2/Dx1Dx2). A CI < 1 was considered as having a synergistic effect. Apoptosis was observed after 24 h following drug administration when the morphology of apoptotic cells became clearly visible. The experimental set-up was as follows: (1) control group (no drug administration), (2) LUT group, (3) OXA group, and (4) combination of the LUT group and the OXA group.

### 2.4. MTT Assay

Cell viability was measured using the 3-(4,5-dimethyl-2-thiazolyl)-2,5-diphenyl-2-H-tetrazolium bromide (MTT) assay. SGC-7901 cells were seeded in 96-well plates. Once cells had grown to approximately 80% confluence, the respective drugs were administered. After 24 h, 20 *μ*L of MTT (0.5 g/L) was added to each well and incubated for another 4 h. The medium was then removed, and the cells were treated with 100 mL of DMSO and shaken for 10 min. The absorbance of MTT formazan was measured at a wavelength of 490 nm using an ELISA reader (Biopeony, Beijing, China). Cell survival rate = [experimental group D (490)/control group D (490)] × 100%.

### 2.5. Hoechst 33258 Fluorescent Staining

A 1 × 10^6^ cells/mL suspension of SGC-7901 cells was prepared, and 1 mL of cell suspension per well was inoculated into a 6-well plate on sterilized coverslips, in triplicates for each group. After 24 h following drug administration, the cells were collected and washed three times with phosphate-buffered saline (PBS). Then, the cells were fixed with 0.5 mL of 10% formaldehyde at 4°C for 15 min, after which the fixator was removed and the cells were washed three times with PBS. The cells were stained with 0.5 mL of Hoechst 33258 solution for 3 min at 25°C. The morphological changes in the apoptotic cells were observed directly under a fluorescence microscope, and images were acquired using the Image Advanced 3.2 System.

### 2.6. Flow Cytometry for Cell Cycle Detection

A suspension of SGC-7901 cells in the logarithmic growth phase was prepared at 1 × 10^6^ cells/mL. The cells were seeded in a 6-well plate at 1 mL per well. After drug administration, the cells were collected and centrifuged at 1000 rpm for 5 min. The supernatant was discarded, and the cells were washed with 1 mL of PBS. Precooled 70% ethanol solution was added, and the cells were incubated at 4°C for 12 h. The cells were centrifuged and the ethanol was discarded, after which the cells were washed twice with PBS. The PBS was discarded, and 1 mL of propidium iodide (PI) solution was added at 37°C. The cells were resuspended after incubation for 30 min in the dark and subjected to flow cytometry within 30 min. The experiment was repeated three times.

### 2.7. Cyclin D1 Assay

SGC-7901 cells were seeded in six-well plates. Following drug administration, cells were washed twice with PBS and then homogenized. The homogenate was centrifuged at 5000 rpm for 10 min. The level of cyclin D1 activity and the standard content were determined using specific detection kits according to the manufacturer's instructions.

### 2.8. Flow Cytometry for Apoptosis Detection

A suspension of SGC-7901 cells in the logarithmic growth phase was prepared at 1 × 10^6^ cells/mL. The cells were seeded in a 6-well plate at 1 mL per well. After drug administration, the cells were collected and centrifuged at 1000 rpm for 5 min. The supernatant was discarded and the cells were collected, centrifuged at 4°C, and washed twice with precooled PBS. Binding buffer (200 *μ*L) was added to resuspend the cells, and 10 *μ*L of Annexin V-fluorescein isothiocyanate was added. The cells were incubated in the absence of light for 15 min. Next, 300 *μ*L of binding buffer and 10 *μ*L of PI were added and the cells were subjected to flow cytometry within 1 h. The experiment was repeated three times.

### 2.9. Western Blot Analysis

Cells were seeded in six-well plates. After treatment, cells were washed twice with PBS and then lysed with cell lysis buffer at 4°C for 30 min. Samples were centrifuged at 12,000 rpm for 10 min, and the supernatant was collected. Protein quantification, SDS-PAGE separation, and blotting on a polyvinylidene fluoride (PVDF) membrane were carried out. Membranes were blocked in 5% milk in PBS with 0.1% Tween-20 at room temperature for 1 h, then incubated with primary specific antibodies against pro-caspase-3, Cyt c, and Bax (1 : 500) as well as cleaved-caspase-3 and Bcl-2 (1 : 300), and glyceraldehyde-3-phosphate dehydrogenase (GAPDH) (1 : 1000) overnight at 4°C. Signals were revealed using the appropriate secondary peroxidase-conjugated antibodies, and the bands were visualized by chemiluminescence using a ChemiDoc XRS System (Bio-Rad, Hercules, CA, USA). The ImageJ software was used to quantify the results of the western blot. GAPDH was used as an internal control.

### 2.10. Statistical Analysis

We used Student's *t*-tests with the program GraphPad Prism (version 5.0; GraphPad Software, San Diego, California, USA) and one-way analysis of variance for multiple comparisons to determine the significance of the differences between the two groups. The data were represented as mean ± standard deviation, and each experiment was repeated three times. A value of *p* < 0.05 was considered statistically significant.

## 3. Results

### 3.1. Effects of LUT and OXA on the Viability of SGC-7901 Cells

**Figure 2:**
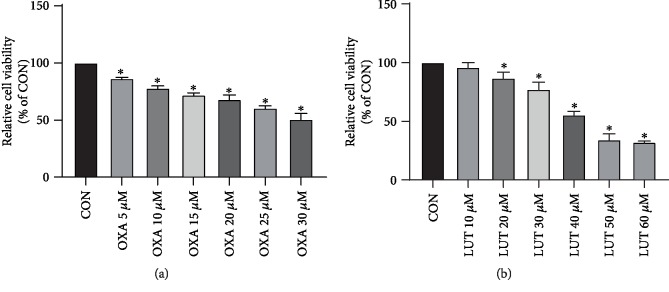
Effect of different concentrations of OXA and LUT on gastric cancer cell proliferation assessed via MTT. ^∗^*p* < 0.05 compared to the control group; CON: control; OXA: oxaliplatin; LUT: luteolin.

The effect of different concentrations of LUT and OXA on the survival of SGC-7901 cells measured via the MTT assay is shown in Figure 2. As indicated, treatment with LUT and OXA for 24 h significantly affected the cell viability of SGC-7901 cells. After careful calculations, we selected LUT (40 *μ*M) and OXA (30 *μ*M) as the subsequent experimental dose and combined dose.

### 3.2. Effects of LUT, OXA, and Their Combination on the Viability of SGC-7901 Cells

**Figure 3:**
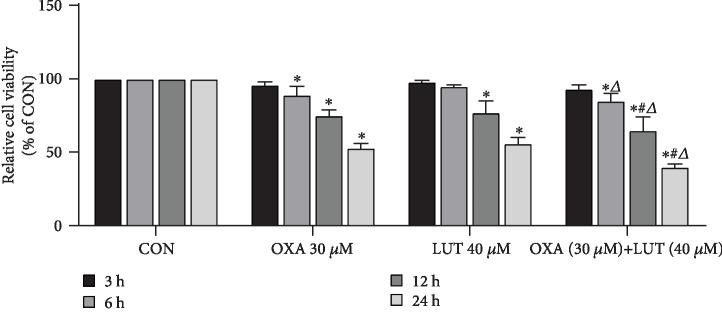
Effect of different drugs and treatment times on gastric cancer cell proliferation assessed via MTT. ^∗^*p* < 0.05 compared to the control group; ^#^*p* < 0.05 compared to OXA; ^*Δ*^*p* < 0.05 compared to LUT; CON: control; OXA: oxaliplatin; LUT: luteolin.

As indicated in Figure 3, with the increase of time, the inhibitory ability of LUT and OXA on cell viability became more and more pronounced when compared to the control group (*p* < 0.05). This effect was most significant at 24 h. Moreover, the effects of the treatment with the two drugs combined were found to be stronger than their individual effects (*p* < 0.05).

### 3.3. Morphological Observation of Apoptosis

**Figure 4:**
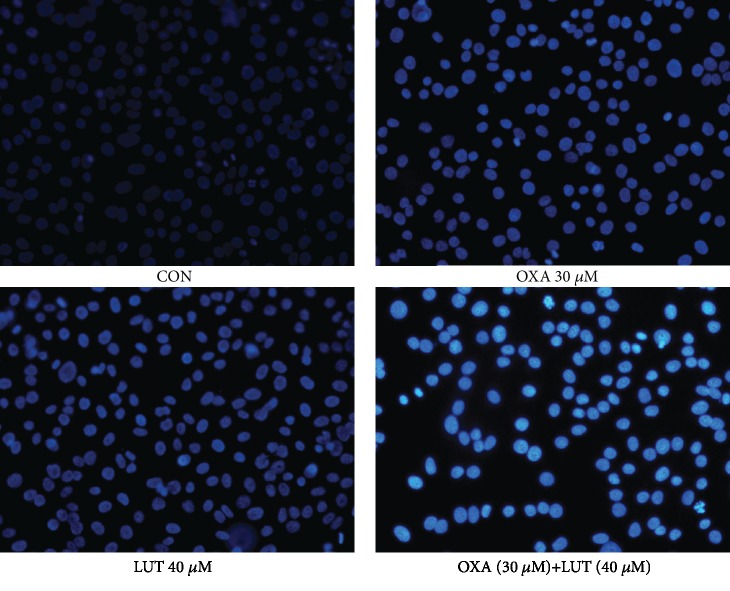
Cellular morphology observation (Hoechst33258, ×200). CON: control; OXA: oxaliplatin; LUT: luteolin.

To investigate the effect of the drugs on cell apoptosis, Hoechst 33258 staining was carried out (Figure 4). After Hoechst 33258 staining, the nuclei of normal cells showed a diffuse blue coloration, whereas the nuclei of apoptotic cells were densely stained or fragmented and bright in color. Apoptosis was observed after cells were treated with LUT, OXA, and a combination of the two drugs. Apoptosis was especially prominent after the combination treatment, while the control cells showed normal nuclear morphology.

### 3.4. Administration of LUT, OXA, and a Combination of the Two Blocked Cell Progression

**Figure 5:**
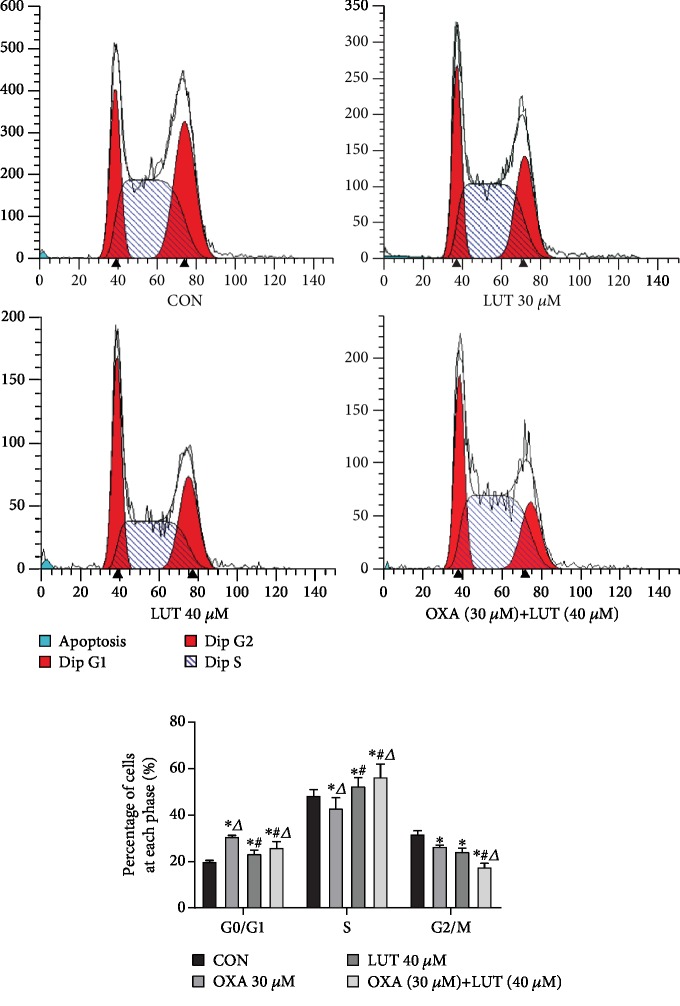
Effect of different drugs on gastric cancer cell cycle assessed by flow cytometry. ^∗^*p* < 0.05 compared to the control group; ^#^*p* < 0.05 compared to OXA; ^*Δ*^*p* < 0.05 compared to LUT; CON: control; OXA: oxaliplatin; LUT: luteolin.

After SGC-7901 cells were treated with each drug or a combination of the two drugs for 24 h, they were subjected to cell cycle progression analysis (Figure 5). Compared with the control cells, those treated with LUT, OXA, or a combination thereof exhibited an increase in the percentages of cells in the G0/G1 phase to varying degrees (*p* < 0.05). Moreover, the combined treatment with LUT and OXA induced the most significant change (*p* < 0.05). These results indicated that LUT, OXA, and combination treatment blocked cell progression in the G0/G1 phase and induced apoptosis.

### 3.5. Expression of a Cell-Cycle-Associated Protein

**Figure 6:**
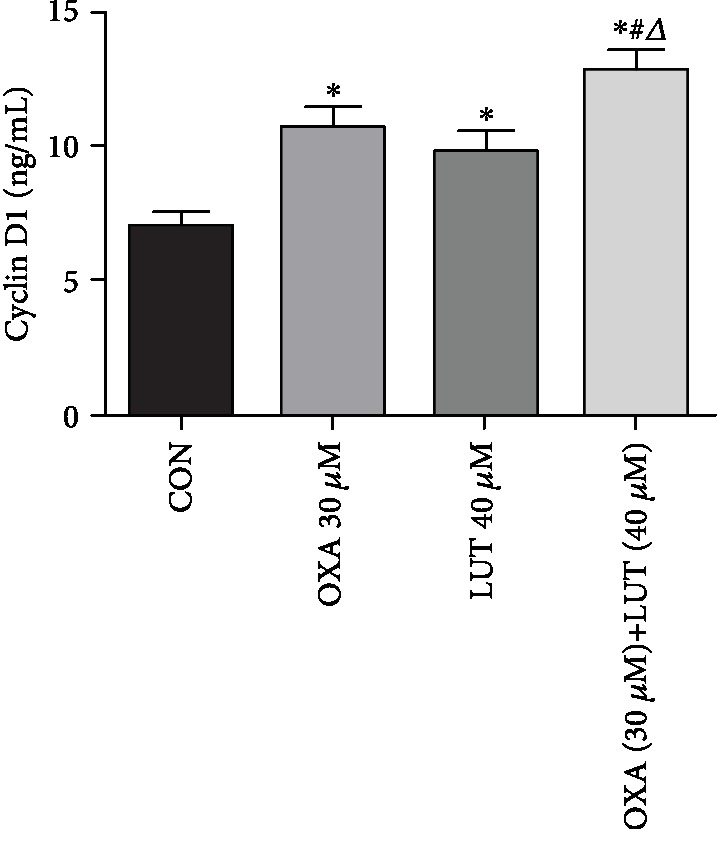
Effect of different drugs on gastric cancer cell expression of the cyclin D1 protein. ^∗^*p* < 0.05 compared to the control group; #*p* < 0.05 compared to OXA; ^*Δ*^*p* < 0.05 compared to LUT; CON: control; OXA: oxaliplatin; LUT: luteolin.

The cell-cycle analysis results revealed LUT, OXA, and a combination of the two blocked cell progression in the G0/G1 phase. It has been suggested that blockage of cell progression in the G0/G1 phase can increase the expression of cyclin D1 [19]. Therefore, we measured cyclin D1 expression in each experimental group (Figure 6). After administration of OXA and LUT, the cyclin D1 levels were significantly increased, when compared to the control group (*p* < 0.05). Moreover, pretreatment with a combination of LUT and OXA significantly increased cyclin D1 levels (*p* < 0.05). These results also confirmed that LUT, OXA, and their combination can arrest the cell cycle in the G0/G1 phase.

### 3.6. Effect of LUT, OXA, and Their Combination Induces SGC-7901 Cell Apoptosis

**Figure 7:**
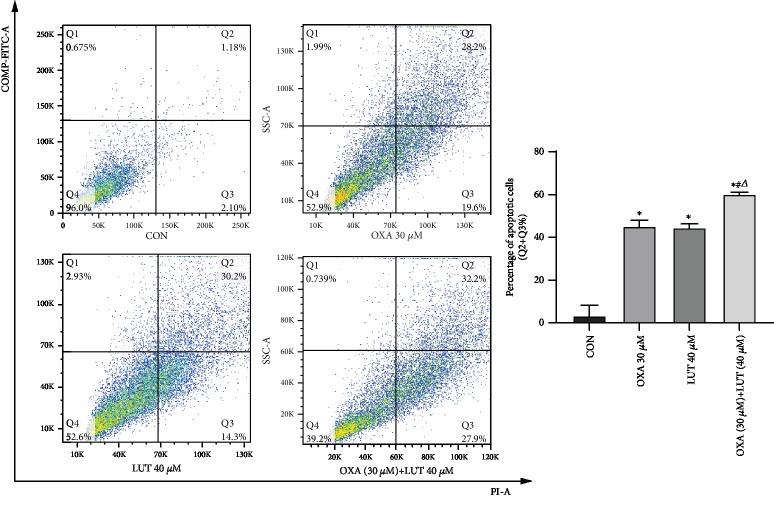
Effect of different drugs on gastric cancer cell apoptosis assessed by flow cytometry. ^∗^*p* < 0.05 compared to the control group; #*p* < 0.05 compared to OXA; ^*Δ*^*p* < 0.05 compared to LUT; CON: control; OXA: oxaliplatin; LUT: luteolin.

Apoptosis was detected after cells were subjected to LUT, OXA, or combination treatment for 24 h. Compared to the control group, the early apoptotic rate after treatment with LUT, OXA, and a combination of drugs was increased (*p* < 0.05). Moreover, the combined treatment induced the most significant changes (Figure 7).

### 3.7. Expression of Apoptosis-Associated Proteins

**Figure 8:**
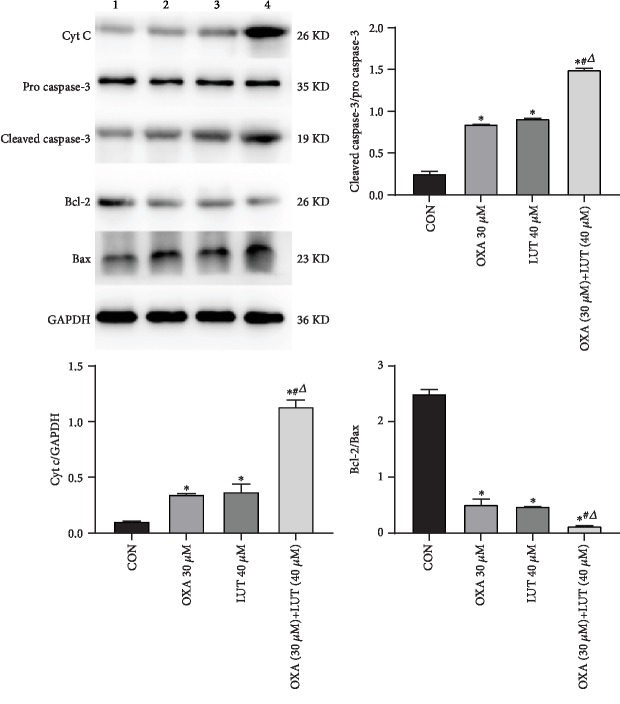
Effect of different drugs on gastric cancer cell expression of apoptosis-associated proteins assessed by western blot. 1: CON; 2: OXA 30 *μ*M; 3: LUT 40 *μ*M; 4: OXA (30 *μ*M) + LUT (40 *μ*M); ^∗^*p* < 0.05 compared to the control group; ^#^*p* < 0.05 compared to OXA; ^*Δ*^*p* < 0.05 compared to LUT; CON: control; OXA: oxaliplatin; LUT: luteolin.

We assessed the protein expression of several crucial regulators of caspase expression (Figure 8). Analysis by western blot showed that when LUT and OXA were administered alone, the expression levels of Cyt c, cleaved caspase-3, and Bax were upregulated and those of Bcl-2 and pro-caspase-3 were downregulated. The effects of the combination treatment with LUT and OXA were more pronounced than those obtained from treatment with LUT and OXA alone (*p* < 0.05).

## 4. Discussion

Common antitumor drugs such as oxaliplatin are reliable for the clinical treatment of gastric cancer. However, long-term use of oxaliplatin can result in nephrotoxicity [20], ototoxicity [21], neurotoxicity [22], and myelosuppression [23]. Moreover, the resistance of tumor cells to oxaliplatin reduces the efficacy of the drug and is a major cause of chemotherapy failure [24]. In recent years, research has increasingly focused on the synergistic effect of traditional chemotherapeutic drugs and high-efficiency Chinese medical ingredients with low toxicity. This combination ensures that while the dose of chemotherapeutic drugs is reduced, it is still sufficiently effective to kill tumor cells. Meanwhile, the toxic and side effects of the drugs are reduced and drug resistance is delayed. Natural plant extracts and their flavonoids have exhibited anticancer properties both *in vivo* and *in vitro*. LUT, a flavonoid that is widely found in many natural plants such as honeysuckle, exhibits many biological effects such as free radical scavenging [25], as well as anti-inflammatory [14] and antitumor properties [16]. The antitumor activity of LUT has been investigated in diverse cancer models [26]. LUT exerts cytoprotective effects against lipid peroxidation products and led to apoptosis of PC12 cells by inducing the phosphorylation of ERK1/2, JNK, and P38 MAPK signal transduction [27]. Moreover, it induced apoptosis by inhibiting PI3K/AKT and ERK1/2 MAPK intracellular signaling and increasing the levels of apoptotic proteins in gastric cancer BGC 823 cells [28]. Moreover, LUT was shown to function as an enhancer, sensitizing cells to doxorubicin-induced autophagy signaling in human osteosarcoma cells [29].

In this study, SGC-7901 human gastric cancer cells were cultured and treated with different doses of LUT. LUT had a significant inhibitory effect on the growth of SGC-7901 cells treated with concentrations of 40 *μ*M of LUT for 24 h, as demonstrated by the MTT assay. OXA showed a similar effect at a concentration of 30 *μ*M. The combination of the two drugs further enhanced the inhibition of SGC-7901 cell proliferation, indicating a synergistic inhibitory effect of LUT and OXA on human gastric cancer cell proliferation. Huang et al. found that LUT induced the apoptosis of breast cancer MDA-MB-231 cells by arresting the cells in the G0/G1 and G2/M phases. Likewise, we demonstrated by flow cytometry that LUT and/or OXA induced apoptosis in SGC-7901 cells by blocking cells in the G0/G1 phase. Moreover, they significantly increased the levels of cyclin D1. LUT was also shown to alleviate cisplatin-induced nephrotoxicity through its antioxidation effects [30].

Cyt c is released by the mitochondria and is an important factor which promotes apoptosis [31]. Moreover, it is a key factor in the initiation of the apoptosis signaling pathway [32, 33]. Once released, Cyt c forms a complex with Apaf1 and procaspase-9, leading to caspase-9 activation, which further activates effector molecules such as cleaved-caspases-3, finally resulting in programmed cell death. Bcl-2 is located in the mitochondrial membrane [34] and plays a key role in maintaining its integrity. Bax is usually located in the cytoplasm and migrates to the mitochondrial membrane when it is undergoing destructive stimuli and subsequently increases the permeability of the mitochondrial outer membrane. As such, Bcl-2 and Bax inhibit and promote apoptosis, respectively. Therefore, maintenance of the Bcl-2/Bax ratio is a key factor in the induction of apoptosis. The results of the western blot analysis showed that compared to the control, LUT and OXA treatment upregulated the expression of Cyt c, Bax, and cleaved-caspase-3. Moreover, the activation of caspase-3 lead to further cleavage of different substrates, resulting in the amplification of the protease cascade and eventually causing cell death. The combined effects of LUT and OXA were more remarkable than those observed when the drugs were administered alone. In addition, Bcl-2 is an important apoptosis-inhibiting gene, while Bax is the most widely studied proapoptotic protein in the Bcl-2 family. The Bcl-2/Bax ratio determines whether cells enter the apoptotic state. If Bax is dominant, then Bcl-2 is inhibited and apoptosis is induced. Otherwise, Bax is inhibited and cells survive. The results of this study showed that, compared to the control group, LUT and OXA decreased the Bcl-2/Bax ratio to varying degrees and that the mechanism by which they induce apoptosis may be related to the activation of caspase-mediated signal transduction. The regulation of apoptotic proteins ultimately leads to apoptosis.

## 5. Conclusions

In summary, the synergistic activity of LUT and OXA inhibited the proliferation of gastric cancer cells *in vitro.* The present study also elucidated the mechanism by which LUT potentiated the sensitivity of SGC-7901 cells to OXA through the Cyt c/caspase pathway. The effects of LUT on OXA uptake are not only due to the increase in cleaved-caspase-3 and Bax expression but also because of the release of Cyt c from the mitochondria. Based on the critical role of Cyt c in controlling the cell death processes in response to various anticancer therapies, we postulate that LUT may act as a potent chemosensitizer, especially in cancer. In fact, we have observed a similar sensitization effect of LUT for other cancer chemotherapeutic agents, such as doxorubicin [29] and cisplatin [35]. Although further investigations are needed to elucidate the underlying mechanisms of the LUT-induced enhancement of the anticancer effects of chemotherapeutic agents, data from this study provide novel evidence for the potential clinical application for LUT as a chemosensitizer in cancer therapy.

## Data Availability

The data used to support the findings of this study are available from the corresponding author upon request.
